# Application of *In Vitro* Plant Tissue Culture Techniques to Halophyte Species: A Review

**DOI:** 10.3390/plants12010126

**Published:** 2022-12-27

**Authors:** Luísa Custódio, Gilbert Charles, Christian Magné, Gregorio Barba-Espín, Abel Piqueras, José A. Hernández, Karim Ben Hamed, Viana Castañeda-Loaiza, Eliana Fernandes, Maria João Rodrigues

**Affiliations:** 1 Centre of Marine Sciences, Faculty of Sciences and Technology, University of Algarve, Ed. 7, Campus of Gambelas, 8005-139 Faro, Portugal; 2 Géoarchitecture Territoires, Urbanisation, Biodiversité, Environnement, Faculty of Sciences and Techniques, University of Western Brittany, 6 av. V. Le Gorgeu, CS 93837, CEDEX 3, 29238 Brest, France; 3 Group of Fruit Trees Biotechnology, Department of Plant Breeding, CEBAS, CSIC, Campus Universitario de Espinardo, 30100 Murcia, Spain; 4 Centre of Biotechnology of Borj Cedria, Laboratory of Extremophile Plants, BP 95, Hammam-Lif 2050, Tunisia

**Keywords:** salt-tolerant plants, micropropagation, plant biotechnology, caulogenesis, callogenesis, suspension cultures, transgenesis, somatic embryogenesis, biochemical applications

## Abstract

Halophytes are plants able to thrive in environments characterized by severe abiotic conditions, including high salinity and high light intensity, drought/flooding, and temperature fluctuations. Several species have ethnomedicinal uses, and some are currently explored as sources of food and cosmetic ingredients. Halophytes are considered important alternative cash crops to be used in sustainable saline production systems, due to their ability to grow in saline conditions where conventional glycophyte crops cannot, such as salt-affected soils and saline irrigation water. *In vitro* plant tissue culture (PTC) techniques have greatly contributed to industry and agriculture in the last century by exploiting the economic potential of several commercial crop plants. The application of PTC to selected halophyte species can thus contribute for developing innovative production systems and obtaining halophyte-based bioactive products. This work aimed to put together and review for the first time the most relevant information on the application of PTC to halophytes. Several protocols were established for the micropropagation of different species. Various explant types have been used as starting materials (e.g., basal shoots and nodes, cotyledons, epicotyls, inflorescence, internodal segments, leaves, roots, rhizomes, stems, shoot tips, or zygotic embryos), involving different micropropagation techniques (e.g., node culture, direct or indirect shoot neoformation, caulogenesis, somatic embryogenesis, rooting, acclimatization, germplasm conservation and cryopreservation, and callogenesis and cell suspension cultures). *In vitro* systems were also used to study physiological, biochemical, and molecular processes in halophytes, such as functional and salt-tolerance studies. Thus, the application of PTC to halophytes may be used to improve their controlled multiplication and the selection of desired traits for the *in vitro* production of plants enriched in nutritional and functional components, as well as for the study of their resistance to salt stress.

## 1. Introduction

*In vitro* plant tissue culture (PTC) techniques are an important tool in industry, agriculture, and plant breeding, by complementing plant production by, for example, micropropagation, genetic transformation, pathogen eradication, and germplasm preservation. The interest in naturally salt tolerant plants (syn. halophytes) as sources of commercial products is on the rise, especially in the context of soil and water salinization. Halophytes can tolerate salt concentrations that are lethal to 99% of glycophytes and can thrive in diverse saline conditions [1], thus being considered a valuable tool to ensure food security and diversification and have a key role within the context of sustainability and climate change, particularly soil and water salinization and freshwater scarcity for agriculture [2]. Moreover, halophytes are also sources of high-added value products with multiple commercial applications, in pharma, food, and cosmetic industries. PTC can be therefore applied to halophyte species, especially to improve multiplication of those with limited sexual and vegetative propagation, to boost the production of bioactive compounds and for the propagation of endangered/vulnerable species [3]. 

**Figure 1:**
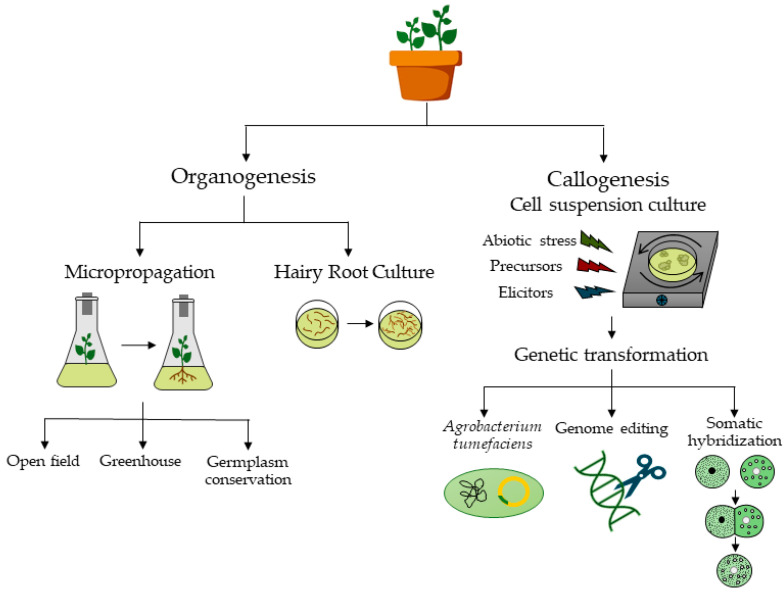
Diagram of methods and applications of in vitro tissue culture applied to halophyte plants.

There are already a considerable number of reports describing the application of PTC techniques to halophyte species, but this information is scattered throughout the literature. Thus, this review provides a comprehensive overview of some general aspects of halophyte plants, their uses, and of the benefits and applications of *in vitro* plant tissue culture (Figure 1). Then, several aspects of the micropropagation of halophyte plants are considered, including material sources and decontamination, micropropagation from axillary buds via node culture, micropropagation via direct and indirect shoot neoformation, caulogenesis—shoot neoformation from callus or cell suspension cultures, somatic embryogenesis, rooting, and acclimatization. Finally, an insight into germplasm conservation and cryopreservation, callogenesis and cell suspension cultures, genetic transformation studies (transgenesis), somatic hybridization, and androgenesis of halophytes is provided (Figure 1).

## 2. Methodology

We consulted the database of PubMed, Web of Science, Embase, and Google Scholar (as a search engine) to retrieve the most updated articles. The keyword “halophyte” was used in combination with, for example, “in vitro culture”, “micropropagation”, “caulogenesis”, “embryogenesis”, “shoot multiplication”, “transgenesis”, “hairy roots”, “regeneration”, “cryopreservation”, “callogenesis”, or “cell suspension”. Only English articles with a full text were considered. The classification as halophytes were confirmed by search in the eHALOPH database, and/or the description of their occurrence in coastal areas.

## 3. *In Vitro* Plant Tissue Culture

PTC techniques have greatly contributed to industry and agriculture in the last 60 years by exploiting the economic potential of medicinal and crop plants [3]. Research on halophytes is increasing and focuses mainly on its biochemical properties and cultivation [1,4,5,6,7,8,9,10,11,12]. The application of tissue culture refers only to a few species [13,14,15,16,17,18], but already yielded the optimization of cosmetic ingredients of high commercial value (CELTOSOME^TM^) from sea fennel and sea holly (*Eryngium maritimum*) [19]. Such techniques are particularly useful for commercial crop species that exhibit limited sexual (seed) and vegetative propagation, which may hamper their large-scale cultivation [20], synthesis of metabolites with commercial interest, and for conservation programs of endangered/vulnerable species. 

Sexual and vegetative propagation are the most common techniques for the cultivation of commercial crops. However, some species can exhibit low rates of seed germination or be difficult to propagate by cuttings, which makes their propagation by such techniques not easy for large-scale commercial exploitation. Some halophytes are already cultivated for commercial purposes, including sea asparagus (*Salicornia* sp.) and quinoa (*Chenopodium quinoa*) for food applications, while others are being considered for cosmetic applications, as for example sea fennel (*Crithmum maritimum*) [21]. But the number of established commercial cultivation methods for halophytes is by far less than for commercial glycophytes. Some of the halophytes with potential commercial applications are not easy to propagate, since germination and vegetative propagation are highly dependent on abiotic factors [21,22,23], therefore *in vitro* PTC techniques are alternative ways to propagate such species, allowing the production of high number of clones, and running as a nursery for producing stock plants for ensuring the supply of high-scale greenhouse cultivation [24,25]. *In vitro* methods allow for the propagation of a high number of plants under controlled environmental conditions, and have several advantages over traditional approaches, including as higher multiplication rates, controlled production and quality, and absence of microorganisms [24,26,27]. 

Plant cell factories (e.g., callus, hairy roots, cell suspensions) are well-established technology platforms for the synthesis of metabolites with commercial interest, providing high-added value plant-derived products that are expensive to synthesize chemically and that naturally occur at low concentrations [28,29,30]. Plant cell factories are currently used to produce ingredients for nutraceuticals, cosmetic, and pharmaceutical products, from different species, such as *Panax ginseng* C.A.Mey., *Taxus* sp., and *Malus domestica* (Borkh.) Borkh. [31]. Plant cell culture technologies can address the challenges for innovation of human nutrition, environment, and commercial uses, allowing to develop science-driven novel products and to create innovative ingredients for the ever-changing consumers’ expectations [28,29,30,31]. These techniques include the establishment of suspension cultures that can be cultivated in bioreactors for large-scale metabolite production under controlled conditions, responding to industry high-quality standards [32]. Likewise, *Rhizobium rhizogenes* (formely *Agrobacterium rhizogenes*) transformed (hairy) roots cultures may be used as an alternative for secondary metabolite production. The main advantage is that hairy roots can produce infinite biomass without growth regulators and synthetize similar or higher amounts of bioactive metabolites than natural roots [33,34,35]. Like cell suspension cultures, hairy roots can also be grown in bioreactors for industrial applications [36]. Moreover, PTC elicitation techniques enable the manipulation of culture conditions to enhance the production of bioactive metabolites with commercial interest [37]. For instance, plants accumulate bioactive metabolites in response to different stress factors, thus, a cell factory can be elicited by the addition of biotic (e.g., proteins, fungus, rhizobacteria, hormones) and/or abiotic (e.g., drought, salinity, light, temperature) elements to the culture medium to enhance the biosynthesis and accumulation of secondary compounds with a commercial interest [38].

The worldwide rapid degradation of ecosystems is leading to a massive loss of plant biodiversity, with high impacts on human livelihoods by negatively affecting food production and natural systems [39]. To reduce these effects, conservation, and management programs for the preservation of threatened species have been developed worldwide, through *in situ* (in natural habitat) and *ex situ* (outside natural habitat) methods that have successfully safeguarded thousands of species [40]. *Ex situ* plant conservation programs traditionally focus on seed banking; however, this is not feasible for some species with, for example, recalcitrant or freeze-sensitive seeds or with few or no viable seeds available. PTC techniques emerged as an important *ex situ* alternative, enabling the propagation of species at risk by using a reduced number of plant materials as initial explants. [40]. *Ex situ* techniques complement *in situ* conservation by supporting conservation programs and were already used for the reintroduction of endangered species into their natural habitats, such as *Cirsium hillii* in Bruce Peninsula National Park (Canada) [41], and the critically endangered species *Rubus humulifolius* that was successfully regenerated after a long-term storage at ultra-low temperatures to *in vivo* conditions in the Botanical Gardens of University of Oulu (Finland) [42].

## 4. Micropropagation of Halophyte Plants

The control of plant micropropagation is a prerequisite for many fundamental studies in genetic or physiology but also for applied purposes such as saline agriculture, site rehabilitation, endangered plant preservation, or secondary metabolites production. It was successfully achieved and reported in relatively few halophytic species from 1991, belonging mainly to the 17 families listed in Table 1, from which the most represented is Amaranthaceae (ex-Chenopodiaceae) with 9 genera, followed by Poaceae (4 genera), Asteraceae and Plumbaginaceae (3 genera each).

### 4.1. Material Sources and Decontamination

Table 1 includes various direct or indirect multiplication protocols starting from different plant sources sampled in nature or already grown *in vitro* in axenic conditions, and comprises basal shoots and nodes, cotyledons, epicotyls, inflorescences, internodal segments, leaves, roots, rhizomes, stems, shoot tips or zygotic embryos.

The establishment of an aseptic culture is a prerequisite for any further experiment in vitro. Most of the protocols cited relate traditional treatments based on the use of ethanol (70–90%), sodium hypochlorite 1–2.5% (Clorox 15–30%, commercial bleach 15–50%), calcium hypochlorite CaOCl_2_ 4%, or mercuric chloride (HgCl_2_ 0.1–0.3%). Some seeds or woody segments may require additional treatments such as the use of various bactericides/fungicides [0.05% Augmentin, 0.1–1% Bavistin, 1–2.5% Benomyl, 0.5% Cuman L, 0.008% Kasugamycin, 0.1% Mancozebe, 0.05–3% Plant Preservative Mixture (PPM), 1% Sovistin, 1% ZeroTol^TM^] for bathing the explants, or are added to the culture media. A surfactant is generally added to the biocide agent (a few drops of tween 20 or 80 or Triton X-100) or used alone as a pre-treatment (Teepol 10%). For *Atriplex* species, seeds are excised from the surrounding bracteoles to eliminate sources of contamination [43]. The sterilization of *Limonium wrightii* includes a pre-treatment of the mother plants with 0.07% Benlate and a bath in Clorox with ultrasonic vibration. Seed disinfection of *C. quinoa* includes a step in a vacuum chamber [44], whereas immature inflorescence of *Diplachne fusca* was flamed for surface sterilization [45].

**Table 1:** Direct and indirect micropropagation experiments derived from buds, adventitious shoots, or somatic embryos reported in halophyte species.

Family/Species	Explant *	Medium **	Treatment	Morphogenic Response	Growth Regulators ***	Best Results	Acclimatization	References
**Acanthaceae**								
*Avicennia marina* (Forssk.) Vierh.	N	MS + AC		Shoot growth	BAP, NAA	5 μM BAP + 1 μM NAA		[46]
		MS + AC		Rooting	IBA	1 μM IBA	+	
	N	MS		Shoot growth	BAP, Kin, IAA	0.5 mg/L BAP + 1 mg/L Kin + 0.25 mg/L IAA		[47]
		MS		Rooting	IAA, IBA, NAA	1 mg/L IBA	+	
**Aizoaceae**								
*Mesembryanthemum crystallinum* L.	H, C, L	MS		Caulogenesis	IAA, BAP, NAA	H, C; 1 μM BAP + 1 μM IAA		[48]
		MS		Rooting		MS PGR free	+	
	H	MS with B5 Vit	80 mM NaCl	Callus induction	2,4-D, Kin	1 μM Kin + 5 μM 2,4-D		[49]
		MS		Somatic embryogenesis	2,4-D, Kin, BAP	2.5 μM Kin		
		MS		Rooting		PGR free	+	
*Sesuvium portulacastrum* (L.) L.	N	MS		Shoot growth	2iP, BAP, Kin, TDZ	40 µM 2iP		[50]
		MS		Rooting	IAA, NAA	5 or 10 µM NAA	+	
	N	MS	0–600 mM NaCl	Shoot growth	BAP, IBA, GA3	200 mM NaCl; 4.44 µM BAP + 0.49 µM IBA + 0.58 µM GA3		[51]
**Amaranthaceae**								
*Atriplex canescens* (Pursh) Nutt.	N	MS/2		Shoot growth	BAP, GA3, NAA	0.01 mg/L NAA + 2 mg/L BAP + 1 mg/L GA3		[52]
	L	MS/2		Caulogenesis (direct)	Kin, 2,4-D	0.01 mg/L 2,4-D + 0.5 mg/L Kin		
		MS		Rooting	IAA, IBA, GA3	0.5 mg/L IBA + 0.1 mg/L GA3	+	
“, *Atriplex torreyi* (S. Watson) S. Watson (syn. *Atriplex lentiformis* ssp. *torreyi*)	Seed, ST	MS, WPM		Shoot growth	2iP	WPM with 5 mg/L 2iP		[43]
		WPM		Rooting		PGR free	+	
*Atriplex gmelinii* C.A. Mey. ex Bong.	H	LS		Callus induction	BAP, NAA	1 μM BAP + 5 μM NAA		[53]
		LS		Caulogenesis	NAA, TDZ	0.1 μM NAA + 20 μM TDZ		
		LS		Rooting		PGR free	n.s.	
*Atriplex halimus* L.	ST	MS/2	0–1000 mM NaCl	Shoot growth	BAP, IBA, GA3, Kin	0.1 mg/L GA3; 200 mM NaCl		[54]
		MS		Rooting	IBA	PGR free	+	
	N	MS		Shoot growth	BAP, Zea, Kin	1 mg/L Kin and BAP		[55]
		MS		Shoot multiplication	BAP, Kin, NAA	0.5 mg/L Kin		
*Beta maritima* L.	In	MS		Shoot multiplication	BAP, IAA, NAA, GA3	1 mg/L BAP		[56]
		MS/2		Rooting	NAA	1 mg/L NAA	+	
*Bienertia sinuspersici* Akhani	S	N6, MS + P	0–200 mM NaCl	Callus induction	2,4-D	MS + P with 1 mg/L 2,4-D, 50 mM NaCl		[57]
		MS + P	CO2, 0–200 mM NaCl	Caulogenesis	BAP	1.2% CO2, 2 mg/L BAP, 200 mM NaCl		
		MS + P	CO2, 0–200 mM NaCl	Rooting	n.s.	1.2% CO2, 50 or 200 mM NaCl	+	
*Chenopodium quinoa* Willd.	H, C, R	MS		Callus induction	2,4-D	Hypocotyl, 0.45 µM 2,4-D		[58]
		MS		Somatic embryogenesis	-	PGR free		
	ST	MS		Shoot growth	Kin, BAP, NAA	1 mg/L Kin + 1 mg/L BAP		[44]
		MS		Rooting	IBA	1 or 2 mg/L IBA	+	
*Halogeton glomeratus* (M.Bieb.) Ledeb.	L	MS		Caulogenesis (direct)	BAP, Kin, NAA	0.5 mg/L BAP + 2 mg/L Kin + 0.2 mg/L NAA		[59]
*Salicornia bigelovii* Torr.	ST	MS		Shoot growth	BAP, NAA	8.89 µM BAP + 0.54 µM NAA		[60]
		MS		Rooting	BAP, NAA	0.44 µM BAP + 10.74 µM NAA	+	
*Salicornia brachiata* Roxb.	ST, N	MS	0–500 mM NaCl	Shoot multiplication	BAP, Kin, IAA, IBA, NAA, 2,4-D	250 mM NaCl; 5.37 μM NAA + 44.4 μM BAP		[61]
		MS/2		Rooting	BAP, NAA	250 or 500 mM NaCl; 5.37 μM NAA + 8.9 or 13.3 μM BAP	+	
	S	MS		Callus induction	2,4-D	2 mg/L 2,4-D		[62]
		MS	80 mM NaCl	Somatic embryogenesis	2,4-D, IBA	0.25 mg/L 2,4-D		
		MS		Shoot growth	_	PGR free	+	
	N	MS * 2		Shoot growth	BAP, Zea	3 mg/L BAP + 0.5 mg/L Zea		[63]
		MS * 2		Shoot multiplication	NAA, TDZ	1 mg/L NAA + 1 mg/L TDZ		
		MS * 2	0–20 g/L MgCl_2_	Rooting	IAA, IBA, NAA	0.5 mg/L NAA + 20 g/L MgCl_2_	+	
*Salicornia europaea* L.	H, ZE, R	MS	170 mM NaCl	Callus induction	2,4-D, TDZ	4.55 µM TDZ		[64]
		MS	0–500 mM NaCl	Caulogenesis	TDZ	4.55 μM TDZ, 170 mM NaCl		
		MS/2 + AC		Rooting	IBA, Kin	2.46 μM IBA + 0.46 μM Kin	n.s.	
	ST	MS	0–100 mM NaCl	Shoot growth	BA, NAA	100 mM NaCl; 0.5 mg/L NAA + 0.5 mg/L BAP		[65]
*Salsola lanata* Pall. (syn. *Climacoptera lanata* (Pall.) Botsch.)	ZE	MS		Shoot multiplication	Kin	2.3 μM Kin		[66]
		MS		Shoot growth	BAP, 2iP, IAA	0.5 μM BAP or 2iP + 0.3 μM IAA		
	L, IS	MS		Callus induction	BAP, Kin, 2iP, IBA, 2,4-D	9 µM 2,4-D		
		MS		Caulogenesis	BAP	8 µM BAP		
		MS		Rooting	BAP, 2iP, IAA	0.5 μM BAP or 2iP + 0.3 μM IAA	n.s.	
*Salsola pestifer* A. Nels. (syn. *Salsola kali* L.)	ZE	MS		Shoot induction	Kin	2.3 μM Kin		[66]
		MS		Shoot growth	BAP, 2iP, IAA	0.5 μM BAP or 2iP + 0.3 μM IAA		
	L, IS	MS		Callus induction	BAP, Kin, 2iP, IBA, 2,4-D	8 µM BAP or 4.9 µM IBA		
		MS		Caulogenesis	BAP	8 µM BAP		
		MS		Rooting	BAP, 2iP, IAA	0.5 μM BAP or 2iP + 0.3 μM IAA	n.s.	
*Sarcocornia ambigua* (Michx.) M.A. Alonso & M.B. Crespo (syn. *Salicornia gaudichaudiana* Moq.)	ST, N	MS	10–30 g/L NaCl, Sediments	Shoot growth	BAP, NAA	ST; 0.5 mg/L NAA + 1 mg/L BAP + 20 g/L NaCl + sediments		[67]
*Sarcocornia fruticosa* (L.) A.J.Scott	N	H&A * 2 with B5 Vit	100 mg/L CNH	Shoot growth		CNH + 100 mg/L Vit		[68]
		H&A *2	150 mg/L Gln, 100 mg/L CNH	Shoot multiplication	BAP, IAA	PGR free + CNH + Gln		
		H&A * 2	Gln, CNH	Rooting	GA3	PGR free + CNH + Gln	n.s.	
*Suaeda edulis* Flores Olv. & Noguez	N	MS		Shoot growth	BAP	1 mg/L BAP		[69]
*Suaeda nudiflora* (Willd.) Moq.	N	MS		Shoot growth	BAP, Kin	1 mg/L BAP + 0.2 mg/L Kin		[70]
		MS, MS/2		Rooting	IAA, IBA, NAA, IPA	MS/2 with IAA + IBA + NAA + IPA (0.5 mg/L each)	+	
**Apiaceae**								
*Crithmum maritimum* L.	ST	B5, MS, WPM	0–300 mM NaCl	Shoot multiplication	BAP, IBA, NAA	MS with 2.5 µM BAP		[71]
		MS		Rooting	IBA, NAA	2.5 µM IBA or NAA	+	
	Shoot	MS		Shoot growth	BAP, IAA, NAA	0.5 mg/L BAP + 0.46 mg/L NAA		[72]
		MS		Rooting	IBA	0.1 mg/L IBA	n.s.	
*Eryngium maritimum* L.	N	MS, MS/2		Shoot growth	BAP, IAA	MS with 1 mg/L BAP + 0.1 mg/L IAA		[73]
		MS, MS/2	1.5–5% Sucrose	Rooting	IAA, IBA, NAA	MS/2 + 1.5% Sucrose + 0.1 mg/L IAA	+	
**Asteraceae**								
*Artemisia caerulescens* L.	Shoot	MS		Shoot multiplication	BAP	1 µM BAP		[74]
*Aster tripolium* L. (syn. *Tripolium pannonicum* (Jacq.) Dobrocz.)	C	MS	0.5 g/L CNH	Callus induction, cell suspen.s.ion	2,4-D, IAA, NAA, BAP, Kin, Zea, 2iP	4.9 µM 2iP		[75]
		MS	0.5 g/L CNH	Caulogenesis	NAA, Kin	5.4 µM NAA + 4.6 µM Kin		
	L	MS	Agar, AgNO3	Caulogenesis (direct)	5.4 µM NAA + 4.6 µM Kin	1.2% agar, 1 g/L AgNO3		
		MS		Rooting	NAA, IBA	27 µM NAA	+	
*Calendula maritima* Guss. (syn. *Calendula suffruticosa* subsp. *maritima* (Guss.) Meikle)	L	MS		Caulogenesis (direct)	BAP, NOA, TDZ, IBA	4.4 µM BAP + 10 µM NOA		[76]
		MS		Rooting	IAA, IBA	1 µM IAA	+	
*Cineraria maritima* Linn.	N	MS with B5 Vit		Shoot growth	BAP, NAA, TDZ	4.54 µM TDZ		[77]
		MS/2		Rooting	IBA	4.92 µM IBA	+	
**Boraginaceae**								
*Mertensia maritima* (L.) Gray	ST, N	MS		Shoot growth	NAA, BAP, Kin, TDZ	4 µM TDZ + 1 µM NAA		[78]
		MS/2		Rooting	IAA, IBA, NAA	4 µM IBA	n.s.	
**Brassicaceae**								
*Crambe maritima* L.	Petiole	MS		Callus induction	BAP, Kin, IAA	0.5 mg/L IAA + 6 mg/L Kin + 1.5 mg/L BAP		[79]
		MS		Caulogenesis	BAP, Kin	6 mg/L Kin + 1.5 mg/L BAP		
		MS		Rooting	IBA, NAA	0.1 mg/L IBA or NAA	+	
**Bromeliaceae**								
*Dyckia maritima* Baker	N	MS		Shoot growth	BAP, Kin	2 μM BAP + 2 μM Kin		[80]
		MS		Rooting	IBA	0.5 μM IBA	+	
**Caryophyllaceae**								
*Honckenya peploides* (L.) Ehrh.	ST, N	MS	0–75 mM NaCl	Shoot growth	BAP, Kin, mT	ST; 25 mM NaCl and 1 mg/L Kin		[81]
		MS	0–75 mM NaCl	Rooting	NAA	25 mM NaCl and 1.5 mg/L NAA	n.s.	
**Ericaceae**								
*Corema album* (L.) D.Don	N	WPM		Shoot growth	2iP, BAP, Kin, mT	2 mg/L 2iP + 1 mg/L Kin		[82]
		Soil		*ex* vitro Rooting	IBA	2 mg/L IBA	+	
**Euphorbiaceae**								
*Excoecaria agallocha* L.	N	MS, WPM, X	Glutathione	Shoot growth	BAP, Zea, IBA	X medium + 13.3 μM BAP + 4.65 μM Zea + 1.23 μM IBA		[83]
		X		Rooting	IBA	0.23 μM IBA	+	
	N	MS		Shoot growth	BAP, Kin, NAA, 2iP	3.9 μM BAP + 1.34 μM NAA		[84]
		MS/2		Rooting	IAA, IBA, NAA	5.41 μM NAA or 2.85 μM IBA	+	
**Fabaceae**								
*Alhagi graecorum* Boiss.	L, P, S	MS	0–200 mM NaCl	Somatic embryogenesis	BAP, TDZ, IAA, IBA	L: 1 μM TDZ + 50 mM NaCl		[85]
	L, P, S	MS		Caulogenesis (direct) + Shoot growth	BAP, TDZ, IAA, IBA	L: 1 μM TDZ + 0.25 μM IAA		
		MS		Rooting		PGR free	+	
*Pongamia pinnata* (L.) Pierre	N	MS		Shoot multiplication		8.8 μM BAP		[86]
		MS/2 + AC		Rooting		PGR free	+	
**Goodeniaceae**								
*Scaevola sericea* (Gaertn.) Roxb.	N	MS		Shoot multiplication	BAP, Kin, NAA	1 mg/L BAP + 0.1 mg/L NAA		[18]
	L, R	MS		Callus formation	2.4-D, BAP, NAA	L; 0.5 mg/L 2,4-D		
	L, R	MS		Somatic embryogenesis	BAP, TDZ	L: 2.5 mg/L BAP; R: 2.5 mg/L TDZ		
		MS/2		Rooting	NAA	2.5 mg/L NAA	+	
**Juncaceae**								
*Juncus roemerianus* Scheele	Seed	MS		Callus induction	BAP, NAA, 2,4-D, CW	2.22 μM BAP + 5.37 μM ANA + 2.26 μM 2,4-D + 5% CW		[87]
		MS		Caulogenesis	BAP, TDZ	13.3 μM BAP		
		MS		Rooting	IAA, IBA, NAA	10.7 μM NAA	+	
*Juncus gerardii* Loisel.	In	MS		Callus induction	BAP, NAA, 2,4-D, CW	2.22 μM BAP + 5.37 μM NAA + 2.26 μM 2,4-D + 5% CW		
		MS		Caulogenesis	BAP, IAA, TDZ	0.44 μM BAP + 0.57 μM IAA		
		MS		Rooting	IAA, IBA, NAA	0.44 μM BAP + 14.8 μM IBA	+	
**Liliaceae**								
*Urginea maritima* (L.) Baker	Bulb scale, L	MS/2, MS		Caulogenesis (direct) + Shoot growth	2.4-D, BAP, Kin, IAA, NAA	Scale: MS + 2 mg/L BAP; L: MS/2 + 2 mg/L BAP + 2 mg/L NAA		[88]
		MS/2		Rooting		PGR free	+	
	Bulb scale	MS		Caulogenesis (direct) + Shoot growth	TDZ	0.55 mg/L TDZ		[89]
		MS		Rooting	IBA	1 mg/L IBA	+	
**Malvaceae**								
*Kosteletzkya virginica* K. Presl ex Gray (syn. *Kosteletzkya pentacarpos* (L.) Ledeb.)	S, ZE	MS		Callus	IAA, Kin	2 mg/L IAA + 1 mg/L 2,4-D		[90]
		MS		Caulogenesis	NAA, Kin	2 mg/L NAA + 1 mg/L Kin		
		MS/2		Rooting	IAA, Kin	0.2 mg/L IAA	+	
**Plantaginaceae**								
*Bacopa monnieri* (L.) Wettst.	L, N	MS, B5		Caulogenesis, shoot multiplication	BAP, Kin, NAA, 2,4-D	Leaf: MS + 1 mg/L BAP 0.25 mg/L Kin		[91]
		MS/2		Rooting	IBA	0.25 mg/L IBA	+	
*Plantago camtschatica* Link (syn. *Plantago depressa* Wild. subsp. *camtschatica*)	ST	MS		Shoot growth	IAA, BAP, Kin, Zea	0.6 μM IAA + 8.9 μM BAP		[92]
	H, C, R, L	MS		Caulogenesis (direct)	IAA, BAP, Kin, Zea	H; 0.6 μM IAA + 8.9 μM BAP		
		MS		Rooting	NAA	0 or 0.5 μM NAA	+	
*Plantago maritima* L.	S	MS		Shoot growth	IAA, BAP, Kin	6.7 μM BAP		[93]
	H, C, R	MS		Caulogenesis (direct)	IAA, BAP, Kin, Zea	Roots; 0.6 μM IAA + 22.8 μM Zea		
		MS		Rooting	IAA, IBA, NAA	0.5 μM NAA	+	
**Plumbaginaceae**								
*Armeria maritima* (Mill.) Willd.	C	MS with B5 Vit	88–118 mM sucrose	Somatic embryogenesis	2.4-D, Kin	4.5 μM 2.4-D + 0.93 μM Kin, 88 mM sucrose		[94]
		MS		Rooting	-	PGR free	+	
*Limoniastrum monopetalum* (L.) Boiss.	ST	MS	0–20 g/L NaCl	Shoot growth	BAP	0.5 mg/L BAP + 0 g/L NaCl		[95]
		MS	0–20 g/L NaCl	Shoot multiplication	BAP, Zea, Kin, 2iP	0.5 mg/L BAP + 5 g/L NaCl		
		MS, MS/2	0–20 g/L NaCl	Rooting	IBA	MS/2 with 1 mg/L IBA	+	
*Limonium bulgaricum* Ančev, *Limonium gmelinii* (Villd.) O. Kuntze, *Limonium latifolium* (Sm.) O. Kuntze, *Limonium meyeri* (Boiss.) O. Kuntze, *Limonium asterotrichum* (Salmon) Salmon, and *Limonium vulgare* Mill.	In	MS		Shoot multiplication	BAP, IBA, GA3	BAP + IBA + GA3 (0.1 mg/L each)		[96]
		MS/2		Rooting	IBA	1 mg/L IBA	+	
*Limonium aureum* (L.) Chaz., *Limonium sinuatum* (L.) Mill., *L. latifolium*	C	MS with B5 Vit	88–118 mM sucrose	Somatic embryogenesis	2.4-D, Kin	4.5 μM 2.4-D + 0.93 μM Kin, 88 mM sucrose		[94]
		MS		Rooting	-	PGR free	+	
*Limonium bellidifolium* (Gouan) Dumort.	C, H, R	MS	58–117 mM sucrose	Somatic embryogenesis	2,4-D, Kin	4.5 µM 2,4-D + 0.5 µM Kin, 117 mM sucrose		[97]
		MS		Plantlet growth	Kin	0.5 µM Kin, 117 mM sucrose	+	
*Limonium bicolor* (Bunge) Kuntze	L	MS		Caulogenesis (direct)	BAP, NAA	4.4 μM BAP + 1.1 μM NAA		[98]
				Rooting	IBA	4.4 μM IBA	+	
*Limonium perezii* (Stapf) F.T.Hubb. ex L.H.Bailey								[99]
*Limonium sinuatum*	L	MS/2		Callus induction	Dicamba, picloram, 2,4-D, NAA	1 mg/L picloram		[100]
	C	MS/2	Gellan gum, agar	Caulogenesis	BAP, TDZ, Zea	1 mg/L Zea, 0.25% gellan gum		
		MS/2		Rooting	-	PGR free	+	
*Limonium wrightii* (Hance) Kuntze	ST, L, In	MS		Caulogenesis (direct)	BAP, NAA	Shoot tips; 8.87 μM BAP + 17 μM NAA		[101]
				Rooting	IBA, NAA	4.92 μM IBA	+	
*Plumbago zeylanica* L.	ST, In	MS		Callus induction	BAP, IAA, IBA, NAA	2 mg/L BAP + 1.5 mg/L IAA		[102]
		MS		Caulogenesis	BAP, IAA, NAA, AdS	0.75 mg/L BAP + 1 mg/L IAA + 1 mg/L NAA + 1 mg/L AdS		
	N	MS		Shoot growth	BAP, IAA, IBA, NAA, AdS	1 mg/L BAP + 0.5 mg/L IBA + 2 mg/L AdS		
		MS, MS/2		Rooting	IAA, IBA, NAA	MS/2 + 0.5 mg/L NAA	+	
**Poaceae**								
*Diplachne fusca* (L.) P.Beauv. ex Roem. & Schult.	In-derived callus	MS		Caulogenesis				[45]
		MS		Shoot multiplication	BAP	1 mg/L BAP		
*Distichlis spicata* (L.) Greene	In	MS		Callus induction	-	PGR free		[103,104]
		MS		Caulogenesis	BAP, NAA, 2,4-D	0.5 mg/L BAP + 1 mg/L NAA + 0.5 mg/L 2,4-D		
		MS		Shoot regeneration	BAP	1 mg/L BAP, then 1 mg/L 2,4-D		
		MS		Rooting	-	PGR free	+	
*Hordeum marinum* Huds.	ZE	MS		Callus induction	CPA, 2,4-D	0.5 mg/L CPA or 2,4-D		[105]
		MS		Caulogenesis	IAA, Zea	1 mg/L IAA + 1 mg/L Zea	+	
*Leymus chinensis* (Trin.) Tzvelev	L, Seed	MS	Glu	Callus induction	2,4-D	Seed; 2 mg/L 2,4-D + 5 mg/L Glu		[106,107]
		MS	2 g/L CNH	Somatic embryogenesis	NAA, Kin	0.2–0.5 mg/L NAA + 2 mg/L Kin		
		MS/2		Rooting	-	PGR free	+	
*Puccinellia distans* (Jacq.) Parl.	Seed	MS		Callus induction	Kin, 2,4-D	2 mg/L 2,4-D + 0.5 mg/L Kin		[108]
		MS, N6		Caulogenesis	Kin, IAA	N6 + 10 mg/L Kin	+	
*Spartina argentinensis* Parodi	L, R, In	MS		Callus induction	BAP, NAA, 2,4-D	L: 0.05 mg/L BAP + 0.5 mg/L 2,4-D; In: 0.01 mg/L BAP + 0.1 mg/L 2,4-D		[109]
		MS		Caulogenesis	BAP, NAA	0.5 mg/L BAP		
		MS		Rooting	NAA	0.5 mg/L NAA	+	
*Spartina patens* Roth) P.M.Peterson & Saarela	S	MS		Callus induction	IAA, 2,4-D	1 mg/L IAA + 1 mg/L 2,4-D		[110]
		MS		Caulogenesis	BAP, IAA	3 mg/L BAP		
		MS, MS/2, MS/4	AC	Rooting	IAA, Kin	MS/4 PGR free	+	
	S	MS		Callus induction	IAA, BAP, BL	0.2 mg/L IAA + 3 mg/L BAP + 0.3 mg/L BL		[111]
		MS		Caulogenesis	IAA, BAP, BL	0.2 mg/L IAA + 3 mg/L BAP + 0.1 mg/L BL		
*Sporobolus virginicus* (L.) Kunth	In	MS		Callus induction	BAP, NAA, 2,4-D, CW	1 mg/L BAP + 1 mg/L NAA + 0.5 mg/L 2,4-D + 5% CW		[112]
		MS		Caulogenesis	BAP, CW	1 mg/L BAP + 5% CW		
**Polygonaceae**								
*Polygonum maritimum* L.	N	MS		Shoot multiplication	BAP, Kin, IAA, NAA	3 mg/L BAP + 0.1 mg/L IAA		[113]
		MS		Rooting	-	PGR free	+	
**Rhizophoraceae**								
*Bruguiera cylindrica* (L.) Blume	H	MS/2 NH4 free		Caulogenesis (direct)	BAP, Kin	PGR free		[114]
				Rooting		PGR free	+	
*Rhizophora annamalayana* Kathiresan	L, ST	MS		Shoot multiplication	BAP, Kin, Zea, CW	ST; 3 mg/L BAP + 3 mg/L Kin + 1% CW		[115]
**Ruppiaceae**								
*Ruppia maritima* L.	Rh	MS/2		Shoot multiplication	BAP, Kin, 2iP, Zea, TDZ	10 mg/L Kin or 20 mg/L 2iP + 1 mg/L NAA		[116,117]
**Salvadoraceae**								
*Salvadora persica* L.	N	MS		Shoot growth	BAP, AdS	8.88 μM BAP + 25 mg/L AdS		[118]
				Shoot multiplication	BAP, Kin, NAA	1.11 μM BAP + 1.16 μM Kin + 0.54 μM NAA		
				Rooting	IBA, NOA	2460.27 μM IBA + 494.56 μM NOA	+	

* Explant sources: BN—basal node; BS—basal shoot; C—cotyledon; Ep—epicotyl; H—hypocotyl; In—inflorescence; IS—internodal segment; L—leaf; N—node; R—root; Rh—rhizome; S—stem; ST—shoot tip; ZE—zygotic embryo. ** Basal medium—B5—Gamborg medium [119]; H&A—Hoagland and Arnon medium [120]; MS—Murashige and Skoog medium [121]; WPM—woody plant medium [122]. *** Growth regulators: 2iP—2-Isopentenyl adenine; 2,4-D—2,4-Dichlorophenoxyacetic acid; 2,4,5-T—2,4,5-Trichlorophenoxyacetic acid; AdS—Adenine sulfate; BAP—6-Benzylaminopurine; CNH—Casein hydrolysate (casaminoacids); CPA—4-Chlorophenoxyacetic acid; CW—Coconut water; GA3—Gibberellic acid; Gln—Glutamine; Glu—Glutamic acid; IAA—Indole-3-acetic acid; IBA—Indole-3-butyric acid; IPA—Indole-3-propionic acid; Kin—Kinetin; NAA—1-Naphtaleneacetic acid; PGRs—Plant growth regulators; TDZ—Thidiazuron (1-phenyl-3-(1,2,3-thiadiazol-5-yl) urea; Zea—Zeatin.

When starting from seeds, problems of dormancy may also require additional treatments such as scarification with sulfuric acid (H_2_SO_4_) [90,123], seed coat removal [96] or longitudinal cut for *Distichlis spicata* [104]. The seeds of *Limonium* species were stratified in sealed and moistened plastic bags at 4 °C during 45 days before decontamination [96]. Growth regulators such as kinetin (Kin) and gibberellic acid (GA_3_) promote seed germination for *Plantago* species [92,93]. This step was differently optimized depending on the species, as for example, the seeds of *Sarcocornia fruticosa* are germinated on Hoagland and Arnon (H&A) medium adjusted at pH 7.2 in the presence of 2% NaCl and 1% agar [68]. Khan and Gul [124] have reviewed the influence of environmental conditions, such as temperature and various chemicals to alleviate salinity effects or innate dormancy on halophytes’ seeds germination, whereas Gul et al. [125] considered the influence of salt, temperature, and light, including considerations on the variability of habitats and the phenomenon of seed heteromorphism.

### 4.2. Micropropagation from Axillary Buds via Node Culture

The common procedure for plant micropropagation involves the multiplication of shoots by the repeated formation of axillary branches. Most of the protocols reported here are initiated from nodal or apical cuttings, i.e., with a meristematic zone. Shoot tips are frequently used as initial explants, already actively growing and easier to decontaminate when used as starting material. Many studies also evaluate the position of nodal explants, i.e., median or basal zone. More specifically, the number of nodes of stem fragments is considered as the main factor for the growth of *Sarcocornia* species [68].

Murashige and Skoog (MS)-based media are predominantly used, with variations such as half- or double-strength concentrations, ammonium (NH_4_) free, or specific composition in macronutrients (X medium for *E. agallocha*) [83], but few species (*A. torreyi* or *C. album*) achieve better results on woody plant medium (WPM) or on Hoagland and Arnon (H&A) medium (e.g., *S. fruticosa*). The carbohydrate source most frequently added is sucrose at 2 or 3%. A higher concentration is frequently preferred for somatic embryogenesis but was only investigated by Aly et al. [94,97] and proved to be favorable at 4% sucrose for *L. bellidifolium*. The medium is generally solidified, from semi-solid to hard, with a gelling agent, mostly agar at 0.8% (0.5–1.0%). The gelling agent is also a source of nutriments and may act not only through the strength of the gel. Gelrite 0.25–0.4% was also used for *L. sinuatum, B. sinuspersici,* and *A. canescens*, generally to avoid the development of basal callus or hyperhydricity. Alternatively, micropropagation was achieved on liquid medium for *P. zeylanica* [102] or *S. ambigua* with a liquid MS medium enriched with natural sediment [67]. A temporary immersion system provided better results for shoot number and size, and better rooting for *M. maritima* [126]. Inversely, shoots of *C. album* produced in temporary immersion bioreactor showed higher vitrification [82]. 

*In vitro* plants are generally cultivated in test tubes for individual analyses, but bigger vials are also used for higher rates of production. Specific devices may also improve the growth and rooting of several species, such as vented lids for higher gas exchange and reduced hyperhydricity [43]. The aeration may increase the growth of the shoots with a better circulation of the sap and the nutriments but also the elimination of gaseous hormones such as ethylene. The procedure is improved by using plant growth regulators (PGRs), mainly cytokinin, for the proliferation of axillary buds inducing multiple shoot formation. Several PGRs combinations were evaluated for inducing a better shoot growth or proliferation of axillary buds. Such a result is frequently achieved using 6-benzylaminopurine (BAP), the most cited cytokinin, rarely alone (*Suaeda*, *Crithmum*), more generally in combination with an auxin, mostly naphthaleneacetic acid (NAA), but also with another cytokinin, such as Kinetin (Kin) or Zeatin (Zea). Some species are successfully propagated in the presence of thidiazuron (TDZ) (*Cineraria*, *Urginea*) or isopentenyl adenine (2iP) (*Sesuvium*, *Atriplex*). At this stage, the addition of GA_3_ was reported for some species of various genera, namely *Sesuvium*, *Atriplex*, *Eryngium* or *Limonium*. Other compounds were successfully added, such as hydrogen isocyanide (CNH), glutamine (Gln), or coconut water (CW) for a complementation in organic nitrogen, various other nutriments, and growth factors. Less used is the addition of activated charcoal, a possible solution to adsorb inhibitory compounds and counteract the negative effects of toxic metabolites and phenolic exudates. It proved to be a key component in the multiplication medium for *S. edulis* [69] or *A. marina* [46]. 

Concerning many salt-tolerant plants considered in this review, the effect of NaCl on shoot multiplication was also examined. It was not required as a medium component for many salt-tolerant species and it gradually decreases the shoot proliferation in species such as *C. maritimum* [71]. Inversely for *L. monopetalum*, the specific addition of NaCl in the basal medium improved shoot proliferation up to 5 g/L [95]. Higher optimal concentrations of 100 or 200 mM are reported for *A. halimus*, for instance (*in vitro* shoots tolerate up to 600 mM) [54], or even 250 mM for a euhalophyte such as *S. brachiata* [61]. Aldahhak et al. [127] published a previous work on *A. halimus* with a small review on micropropagation conditions including three other species: *A. nummularia*, *A. glauca*, and *A. canescens*.

The only example of a seagrass presented in this review is the species *Ruppia maritima*. The principles of micropropagation are universal, but each species may need special requirements depending on its life cycle, morphology, and habitat. Seagrasses include over 50 species, mainly Poaceae, living in sea water or estuaries, generally submerged. They require specific strategies for decontamination and are cultivated in liquid media made with artificial seawater. These special macrophytes are frequently compared to algae when analyzing their responses to different culture media and additives. Terminal rhizome segments of *R. maritima* were decontaminated after several treatments with fungicide (Captan 2.5 g/L for 24 h), Clorox, and a final soak with antibiotics (erythromycin + rifampicin). Rhizome fragments were placed in culture tubes submerged with liquid medium (synthetic seawater with half-strength (MS/2) and 1% sucrose) under high illumination. Single-node explants did not develop but only apical zones. All cytokinins tested improved the rhizome development (BAP, Zea, Kin, TDZ and 2iP) but only 2iP, a cytokinin present in seawater and sediments, induced a dose-dependent response. Rooting was not stimulated by NAA [116]. 

### 4.3. Micropropagation via Direct Shoot Neoformation

Direct neoformation was observed in few halophytic species. The initial explant should be devoid of meristematic tissue and requires generally the dedifferentiation of somatic cells to organize a new meristematic zone. These protocols are considered conform and are supposed to avoid somaclonal variation events, similar to microcuttings with buds. They offer new possibilities of plant multiplication and plant breeding when associated with mutagenesis or transgenesis.

*M. crystallinum* plants have been regenerated from hypocotyls and proximal half of cotyledons, excised from 14-day-old seedlings, placed on MS medium with BAP and indole-3-acetic acid (IAA) [48]. Multiple shoot regeneration occurred when hypocotyl explants were placed horizontally in full contact with the medium. Hypocotyls of 4-week-old seedlings proved also to be the most efficient for adventitious shoot regeneration of *P. camtschatica*. Regeneration was high with BAP and Kin but spontaneous rooting occurred only in the presence of Zea [92]. A propagation protocol was developed for a rare tree mangrove, *B. cylindrica*, also based on hypocotyl segments, but originated from viviparous propagules: a modified MS/2 medium devoid of ammonium nitrate (NH_4_NO_3_) produced the highest rate of direct caulogenesis and was improved during monsoon [114]. An efficient protocol was established for plant multiplication by direct organogenesis from leaves of endemic *C. maritima*. Shoot buds appeared at the cut surface of leaves on MS medium, with BAP alone or in combination with 2-Naphthoxyacetic acid (NOA) [76]. Similar result was observed from leaf explants (basal and medium parts) of *L. wrightii* on MS medium with a combination of BAP and NAA [101].

### 4.4. Micropropagation via Indirect Neoformation

This process requires a first step of callus induction initiated from explants excised from various organs, including limb, petiole, stem, hypocotyl, or root, and more rarely meristematic cells, such as young inflorescences or apical zones which may be finely cut to promote callus induction. The explants are cultivated on usual basal media, generally MS, but MS + Phosphorus, MS/2 or Linsmaier and Skoog (LS) medium was reported once. The basal media are frequently enriched with different PGRs, mostly auxins, such as 2,4-D alone or combined with NAA, IAA, BAP, or Kin. Moreover, TDZ, 2iP or picloram are often used alone to induce callogenesis. Other combinations also proved to be successful, namely BAP/IAA, IAA/Kin, or more complex associations, one of them including brassinolides (Bl) for *S. patens*. All the calli depicted in Table 1 were able to induce newly formed adventitious shoots via caulogenesis, and less frequently via somatic embryogenesis, and generally require a subculture on new medium for microshoots development and rooting, or for embryo maturation and conversion. A few papers reported the regeneration from calli-derived cell suspension cultures.

Indirect caulogenesis was reported in eight families, the Amaranthaceae family being the most represented, but the Poaceae family is also frequently mentioned. However, regeneration via caulogenesis is assumed when the shoots of embryogenic origin, common in this family, is not clearly demonstrated. 

Regarding the Amaranthaceae family, the calli were derived from various explant types and were generally induced by a combination of NAA + BAP or BAP + IBA as for *A. gmelini* or *S. pestifer*, and the shoots emerged after their transfer to media with TDZ + NAA or BAP, respectively [53,66]. Moreover, callus induction occurred with 2,4-D only for *B. sinuspercici* and *S. lanata*, and with TDZ only for *S. europaea*, which illustrates the wide variety of possible treatments in this family [57,66,128]. 

*T. pannonicum* produced callus in the presence of 2iP and shoots developed after the transfer to NAA-Kin [75]. A combination of Kin, BAP, and IAA induced callus from petiole of *C. maritima,* followed by IAA (0.5 mg/L) removal for shoot development [79]. Callogenesis was induced on IAA + Kin, with twice as much auxin as cytokinin, where stem and seeds of *K. virginica* and caulogenesis occurred after transfer to NAA + Kin at a similar ratio higher in auxin content [90]. 

In the genus *Limonium*, small callus developed at the marge of leaf explants with BAP + NAA of *L. bicolor* and shoots were induced without subculture. The process was successfully combined with transformation experiments [98]. In *L. sinuatum*, friable callus was induced also from leaf fragments but with picloram alone, and a fast-growing suspension culture was established. Shoot regeneration was achieved by various cytokinins but especially Zea [100]. In the same family, callus was initiated at the cut end of stems of *P. zeylanica* with BAP + IAA. A high rate of shoot regeneration was observed after transfer with BAP + IAA + NAA (with an unusual higher auxin content) and further increased by 50% when adenine sulfate (AdS) was also added [102]. In the case of monocotyledons, callus was produced from seeds or inflorescences of Juncaceae species with NAA + 2,4-D + BAP, and shoots developed either with BAP alone at a high concentration for *J. roemerianus*, or at a low concentration with a trace of IAA for *J. gerardi* [87]. In the Poaceae family, a callus of *S. argentinensis* developed in the presence of 2,4-D + BAP, their concentration being 5 times higher when applied on leaf explant than on inflorescences [110]. For *S. patens*, callus was induced from seedlings with IAA + 2,4-D [110]. Caulogenesis of both species was induced by BAP alone [109,110], but for *S. patens* the shooting was improved with a combination of BAP, IAA, and Bl [111].

### 4.5. Somatic Embryogenesis 

The process and efficiency of regeneration by somatic embryogenesis is generally influenced by three key factors: a genotype cultivar with the certain regeneration efficiency, explant source, and regeneration medium for the given cultivar [59]. Although vegetative tissues should be considered the ideal alternative explant source for embryogenic callus induction, since they are always available, their use to develop a regeneration platform is much more difficult [106]. 

Medium optimization is also a necessary step to achieve ideal culture conditions that positively influences the in vitro regeneration according to their physicochemical properties, and it usually involves the mix of salts and vitamins, a carbon source, and hormone combinations [129]. The induction of nodular embryogenic calli and embryos is developed in media with an elevated concentration of auxins (2,4-D, NAA), and low levels of cytokinin, mainly 6-benzylaminopurine (6-BA), and Kin, to stimulate embryo development and germination. In general, a significant reduction in the level of auxin promoted embryo germination [62,107]. 

The best example of somatic embryogenesis in halophytes was reported in *S. europaea*, where mature embryos were the best type of explant for callus induction and in vitro regeneration, through short treatment with 2,4-D in mature seeds, and callus induction from hypocotyls in MS medium supplemented with 4.55 μmol/L TDZ for 3–4 weeks after germination. The callus differentiated into somatic embryos with shoots at a 27.60% ratio after subculture with indole-3-butyric acid (IBA), Kin and activated charcoal (AC) [64], as for *H. glomeratus* where its subculture is crucial for callus proliferation and embryogenic callus formation, as well as a low level of 2,4-D, needed for callus differentiation during this step [130]. In addition, a relatively low water content in callus plays a key role in somatic embryo formation and is beneficial for plants [131,132].

### 4.6. Androgenesis

Haploid production is widely used to produce uniform homozygous lines of main crops. It is also a tool of great value for genetic analyses or to induce some genetic changes at haploid level before to fix them after doubling. Haploids may be particularly useful in identifying dominant and recessive genes involved in the various components of the mechanisms of salt tolerance. Kenny and Caligari [133] induced the regeneration of shoots of *A. glauca* from clusters of young flowers used as a starting material. Shoot organogenesis took place directly from microspores and presumed haploid plants and spontaneous diploid plants were successfully rooted but the ploidy status of the plants needs confirmation.

### 4.7. Rooting

Rooting individual microshoots obtained by micropropagation is an indispensable step for all the studies that aim at growing plants in greenhouses, in the field, or in their natural habitats. Root induction may occur spontaneously in the basal or propagation medium, but mostly healthy shoots are excised and transferred to a rooting medium before acclimatization. The MS basal medium, at full, half-, or less frequently double-concentrated, is the most used for rooting, as found for *S. brachiata*, which suggests that shoot multiplication conditions, as well as genotype, may also influence the rooting efficiency [61,62,134]. For instance, Kulpa et al. [81] described how the origin of the shoot (apical > basal) and the type of cytokinin used for shoot multiplication of *H. peploides* had a significant impact on the size and number of roots—meta-Topolin (mT) improved spontaneous rooting when compared to BAP and Kin [81]. Similarly, the shoots of *J. roemerianus* regenerated with BAP supplementation induced the production of adventitious roots, but not those supplemented with TDZ [87]. In turn, Kin was found to be the most effective for spontaneous rooting of adventitious shoots of *P. camtschatica* [92]. Moreover, transferring the *in vitro*-produced shoots to a basal medium free of growth regulators is the most efficient method for rooting numerous species belonging to Amaranthaceae and Poaceae families. 

When rooting does not occur spontaneously or after subculture on a PGR-free medium, the main factors influencing the root induction are auxin type and concentration: IBA is the most frequently used, followed by NAA and IAA, mainly alone but also in combination, or associated with BAP, Kin or GA_3_. The rooting efficiency was generally higher on medium containing low auxin concentrations to avoid inhibition of root growth and basal callus development [71]. For *C. quinoa*, the requirement for IBA is cultivar-dependent, but the most effective technique is the *ex vitro* rooting without any rooting treatment [44]. For *C. album*, rooting proved to be a difficult process as for many other woody species, and only *ex vitro* conditions in perlite/vermiculite after a dip in concentrated IBA (2 g/L) were partially successful [82]. 

Additives such as CNH, Gln, myo-inositol, glycine, AC, ascorbic acid, carbon dioxide (CO_2_), NaCl, and magnesium chloride (MgCl_2_) are amongst the most efficient rooting media, being added as nutriments, elicitors, antioxidants, or in studies of salt requirement or tolerance. For example, the best rooting of *S. brachiata* microshoots occurred in the presence of 250 up to 500 mM NaCl [61]. Another study with *S. brachiata* showed that the addition of 20 g/L MgCl_2_ to double-concentrated MS medium with 0.5 mg/L NAA significantly improved the rooting efficiency [63], whereas *S. europaea* rooted better on half-strength MS with activated charcoal and a combination of IBA and Kin [64]. During *in vitro* rooting of *L. monopetalum*, the tolerance to NaCl concentrations up to 10 g/L was observed but the root number was reduced as the NaCl concentration increased [96]. The effect of sucrose content on *Eryngium* species was analyzed, and *E. maritimum* had the highest root number with MS/2 with 1.5% sucrose and 0.1 mg/L IAA [73].

The firmness of the rooting medium may also play a key role in root induction. For example, *L. sinuatum* rooted in a hard medium with 5 g/L gelrite [100], *A. canescens* with 4 g/L [52], and *H. glomeratus* on a semi-solid medium with 4.5 g/L agar [59], whereas *S. nudiflora* was rooted in liquid medium [70]. *M. maritima* shoots placed in a temporary immersion system (TIS) produced more developed roots and leaves and high acclimatization performances [126].

### 4.8. Acclimatization

A complete process for plant micropropagation requires the control of the acclimatization and hardening of the in vitro-produced plants, but some papers do not describe this crucial step. Rooted shoots need to be carefully washed to avoid contaminations before being placed in suitable substrates to ensure good aeration and a high humidity level. The most frequently used substrates consist of a mixture of various components, which are occasionally also used alone: vermiculite, perlite, peat moss, peat pellets (Jiffy), sand, or soil. In some reports, the substrate is autoclaved, and fungicide is sprayed to avoid losses by fungal contaminations. Most of authors cover the pots with clear plastic film or a lid to maintain the relative humidity high, which is gradually removed over a 2-week period. The plants are irrigated with water sterilized or not, or with diluted macronutrients (MS/2 salts or Hoagland’s nutrient solution or commercial fertilizers). Overall, the acclimatization step is successfully achieved for various species after the selection of vigorous rooted shoots, with survival rates varying between 55 and 80% or more. Exceptionally, further improvement is still required for some species, such as *P. camtschatica* with only 27% of survival [92].

## 5. Germplasm Conservation and Cryopreservation

The application of these micropropagation techniques may also contribute to the long-term preservation of germplasm through the cryopreservation process. Small propagules (any structure able to develop a full organism—buds, somatic embryos, embryogenic calli) are generally encapsulated in alginate, treated with cryoprotectants, and dehydrated, allowing vitrification of internal solutes without formation of ice crystals and disruption of cell membranes during the cooling process. Many *Limonium* species are of great interest for their ornamental use, although they unfortunately are increasingly threatened by human activities. The opportunity for long-term conservation of *Limonium* genetic diversity was developed with a Sicilian genotype of *L. serotinum*, where in vitro shoot tips were successfully cryopreserved using the droplet-vitrification technique [135].

## 6. Callogenesis and Cell Suspension Cultures

Several publications reported in this review aimed at the production of fast-growing callus and/or suspension cultures for fundamental studies of the cellular and molecular basis of salt tolerance, but also for secondary metabolites production (Table 2). Thus, the culture media and the used PGRs are not always compatible with the regeneration process and may induce somaclonal variation, mutations, or changes in the ploidy level. In turn, some other publications described the micropropagation of recalcitrant species that remain blocked at the callus stage with no regenerative capacities to date.

Calli derived from halophyte species can provide a very suitable model for the physiological, biochemical, and molecular analysis of the effect of environmental stresses in plant cells. In general, there is less information about physiological, biochemical, and molecular aspects in halophytic plants than in glycophytic plants due to different reasons, including their long-life cycles, heterozygosity, and its difficulty in establishing in vitro cultures. However, it should be noted that halophytes can serve as model plants to study adaptation mechanisms to environmental stresses, including salinity [75]. Regarding the establishment of *in vitro* culture of halophytic plants, one of the first studies was reported in *S. europaeae* and *S. maritima* [128]. These authors showed callus formation using B5 medium supplemented with 1 ppm IAA and 10 ppm Kin. In addition, the authors reported that even under *in vitro* conditions the growth rate of the calli was much better in the presence of 0.75–1.0% (129–170 mM) NaCl than in their absence (control conditions).

### 6.1. Biochemical Studies

Callogenesis is often induced to produce cell suspension cultures that can be used for biochemical purposes. In that regard, callus formation was established from *A. maritima* in MS medium supplemented with sucrose, 2,4-D, and Kin [153]. These calli were used for the development of an efficient protocol to produce cell suspensions, a prerequisite for further in vitro studies on the production of bioactive specialized metabolites [153].

The callogenesis process has been used to study the effect of salinity on the antioxidant metabolism of halophytes. Yang et al. [161] have studied the effect of salt stress on the response of antioxidants enzymes in *N. tangutorum* calli. These authors observed an increase in enzymes that eliminate hydrogen peroxide (H_2_O_2_) and superoxide (O_2_^−^) (Ascorbate peroxidase (APX), and catalase (CAT) and superoxide dismutase (SOD), respectively)) due to the salinity (0 to 200 mM NaCl), suggesting an important role for these enzymes in salt tolerance of the calli [161]. An increase in CAT and SOD activities as well as in antioxidant capacity was also noticed in callus from the halophyte *S. persica* in the presence of NaCl (0 to 200 mM NaCl) [160], suggesting the use of this halophyte as a source of antioxidants in harsh saline desert conditions for humans (fruits) and cattle (leaves) [160]. A similar response of the antioxidant machinery was reported in callus from the halophyte species *S. baryosma*, *T. triquetra*, and *Z. simplex* [141], which displayed a high antioxidant capability, according to the ferric-reducing/antioxidant power (FRAP) and 2,2-diphenyl-1-picrylhydrazyl (DPPH) activities, suggesting the use of the plant extracts for nutraceutical formulations [141].

### 6.2. Salt-Tolerance Studies

*In vitro* culture provides a controlled and uniform environment for studying physiological and biological processes in plants, particularly at the cellular level under abiotic stress conditions, including salinity [163]. Different works have shown the usefulness of using cell and/or tissue cultures for the evaluation of tolerance to salinity at the cellular level, since these studies require less time, and the environmental conditions are easily controllable. Thus, the callogenesis process is a very important step for salinity tolerance studies of regenerated in vitro plants.

Although the response to salinity at the cellular level and at the plant level may be somewhat different, some studies have shown that the salinity tolerance observed in whole plants is also observed at the callus culture level [164,165]. However, in other cases, a greater tolerance to salinity is observed at the whole plant level than at the callus culture level, as occurred in the facultative halophyte *S. portulacastrum* [50,51]. In this sense, 200 and 400 mM NaCl produced a dramatic decrease in callus growth, water status, and cell membrane damage [50]. However, in whole *in vitro* plants, 400 mM NaCl did not affect plant growth, whereas 200 mM NaCl stimulated biomass accumulation. In this case, the growth of *Sesuvium* seedlings was decreased in the presence of 600 mM NaCl. These differences can be due to the direct phytotoxic ions’ exposure to the calli cells and the inability of callus cultures to distribute toxic salts into different parts because of the dedifferentiated nature of the cells, unlike the whole plant, thanks to its higher level of tissue organization [50,51]. The effect of NaCl addition was studied in *S. persica* L. calli [160]. These authors observed that the presence of NaCl (50 to 200 mM) in the culture media reduced fresh weight but increased the dry weight at moderate NaCl levels. In addition, NaCl increased proline, sugars, and protein contents. These results suggested a cellular tolerance to lower salinity in this halophytic species [160]. Callus cultures were used to evaluate the response of the antioxidant metabolism to NaCl stress in three halophyte species: *S. baryosma*, *T. triquetra*, and *Z*. *simplex*. The callus was cultured on MS medium in the presence or the absence of different NaCl levels (50, 100, and 200 mM) [141]. In the presence of 50 and 100 mM NaCl, an increase in soluble protein content and dry weight was observed, whereas in the presence of the highest NaCl concentration no significant changes were observed for these variables [141]. In another work, the growth of *S. patens* callus, maintained on MS-based medium, was stimulated in the presence of 170 mmol/L NaCl compared to callus grown without NaCl, whereas 340 mmol/L NaCl did not alter growth, which suggests a cellular salinity tolerance for this halophytic species [166]. Complementarily, steady-state fluorescence analysis indicated that plasma membrane rigidity was conserved at the salinity concentrations tested, whereas the abundance of short-chain fatty acids in the plasma membrane suggests that they may play a role in the salt tolerance of cells [166]. In *Cakile maritima* suspension cells, Ben Hamed et al. [147] identified two behaviors in response to salinity—one related to a sustained depolarization due to Na^+^ influx through the non-selective cation channels leading to programmed cell death of these cells, and a second one related to a transient depolarization allowing cells to survive. Arbelet-Bonnin et al. [148] reported the presence of Salt Overly Sensitive (SOS)-like genes CmSOS1, CmSOS2, and CmSOS3 [148]. These SOS-like genes present constitutive expression levels which could be regulated according to the NaCl concentrations. Moreover, the SOS system activation during salt stress seems to be dependent on a ^1^O_2_ (singlet oxygen) production, in which an increase in intracellular calcium initiates the SOS system toward survival [144].

## 7. Genetic Transformation Studies (Transgenesis)

Information on the transformation of halophyte plants is quite scarce due to the lack of transformation systems and/or efficient protocols for the regeneration of the transformed plantlets [167]. In addition, the transformation efficiency of *A. tumefaciens* depends on different factors, including the selection pressure, the bacterial concentrations, as well as the type of plant material used [15,98]. Table 3 summarizes the transgenesis studies performed with halophyte species. 

The first paper on this subject was published in 1999 by Dr. Ken Ishimaru in Japan. This author transformed the vector pBI121, including the β-glucuronidase (GUS) and kanamycin (Km) resistance genes into *M. crystallinum* cells via *A. tumefaciens*. However, when using callus, no transformation results were obtained. The transformation efficiency varied depending on the plant tissue used for transformation, but the best results were obtained from root and hypocotyl tissues, with rates of transformation higher than 50% in both cases [167]. This was a pioneering work in the transformation of halophytic species and opened a door to transform other species as well as to extend our knowledge on the response to salinity in plants. Some years later, Uchida et al. [53] carried out the transformation of *A. gmelini* callus with *A. tumefaciens* cells harboring the pBI121 plasmid. The transformed calluses were selected by GUS expression and histochemical assay, and the presence of the GUS gene was also confirmed by Southern blot. However, the transformation efficiency from calluses was very low (0.02%) [53]. Yuan et al. [98] used shoot explants from *L. bicolor* for the transformation via *A. tumefaciens* harboring the plasmid pTCK303. Some of the regenerated plantlets showed GUS staining as well as positive GUS expression. Based on the polymerase chain reaction (PCR) results, the authors observed a 4.43% transformation frequency [98].

More recently, and using the same halophyte plant model, Hwang et al. [15] described efficient transient transformation protocols using either *A. tumefaciens* or *R. rhizogenes* (syn. *A. rhizogenes*) for different ice plant materials: hypocotyl-derived callus, *in* vitro-grown seedlings, and pot-grown young plants. Concerning callus material, the highest transformation rate (3%) was obtained on 5-day-old calli co-cultured with 2.5 × 10^9^ cfu/mL bacteria containing the T-DNA binary plasmid pBISNI. The transformation rates declined in oldest calli and with higher concentrations of bacteria. On the other hand, the transformation rates were much higher when using *in vitro* young plant seedlings, reaching 85% for 3-day-old plant seedlings. Plant seedlings were also infected with two different strains of *R. rhizogenes* containing the T-DNA binary vector pCAMBIA1303, which led to a 100% transient transformation efficiency from 3- and 5-day-old seedlings. In addition, pot-grown ice plants, 5 to 6 weeks old, were syringe-infected with different *R. rhizogenes* strains, containing the plasmids pRiA4, pRi8196, or pRi1855, respectively, which resulted in 75% of plants containing transgenic roots after 2 weeks of infection [15].

Fang et al. [168] succeeded in cloning, characterizing, and transforming the FLC (FLOWERING LOCUS C) gene, a strong flowering inhibitor, from the halophyte *T. halofila* (ThFLC). Ectopic expression of ThFLC in *Arabidopsis* by using the *Agrobacterium* floral dip method caused a late-flowering phenotype. These authors also engineered an RNAi construct, developed from a 309 bp fragment of ThFLC cDNA, for gene-specific silencing of endogenous ThFLC in *T. halofila*. This resulted in an early flowering phenotype of all lines obtained while maintaining the same salt tolerance as the wild type, providing a good research model for studies of salt tolerance in plants. In addition, the manipulation of the FLC gene can allow us to manipulate the vegetative growth of certain plants of interest [169]. 

Transformation via *R. rhizogenes* is a biotechnological method not classified as a genetically modified organism (GMO) by the European Union. This bacterium induces the growth of hairy roots at the infection sites due to the insertion of a plasmid-borne transfer DNA (T-DNA) [14]. *In vitro* hairy roots are an excellent source for secondary metabolites [165]. In a recent paper, Lokhande et al. [14] transformed in vitro leaf and stem explants from the halophyte *S. portulacastrum* L. Leaf explants showed a higher root induction capability than stem explants [14]. These authors assayed the phytoremediation capability of the induced hairy roots against different textile dyes, observing an efficient degradation activity [14].

*R. rhizogenes*-induced hairy roots were also obtained by transformation of primary leaves of in vitro *N. schobery* L. seedlings [170]. The extracts of these hairy roots revealed a significantly higher content of some secondary metabolites, including flavonoids, hydroxycinamic acid, pectins, sapononins, and catechin, than the control plant roots. In addition, the authors noticed that ethanolic extracts of transformed hairy roots had a high antiviral activity against different influenza virus subtypes [170].

**Table 3:** Transgenesis experiments reported in halophyte species.

Species	Transformed Organ	Gene(s) *	Vector	Procedure	Studied Trait	Reference
**Aizoaceae**						
*Mesembryanthemum crystallinum*	Callus, Seedling	GUS, NPTII	*Agrobacterium tumefaciens*	Co-culture	n.s.	[15]
	Root	GUS	*R. rhizogenes*	Syringe injection	n.s.	
	Seedling	GUS	*A. tumefaciens*	Co-culture	Stress responses	[167]
**Amaranthaceae**						
*Atriplex gmelini*	Callus	GUS	*A. tumefaciens*	Co-culture	n.s.	[53]
*Sesuvium portulacastrum*	Leaf, stem	Ri-TDNA	*R. rhizogenes*	Co-culture	Phytoremediation	[14]
*Suaeda salsa* (L.) Pall.	Hypocotyl	GUS	*A. tumefaciens*	Co-culture	n.s.	[171]
**Brassicaceae**						
*Thellungiella halophila*	Flower	FLC	*A. tumefaciens*	Floral dip	Flowering	[168]
**Nitrariaceae**						
*Nitraria schoberi* L.	Primary leaf	Ri-TDNA	*R. rhizogenes*	Co-culture	Anti-influenza activity (H5N1, H3N2)	[170]
**Plumbaginaceae**						
*Limonium bicolor*	Leaf segment	GUS	*A. tumefaciens*	Co-culture	n.s.	[98]
**Poaceae**						
*Leymus chinensis*	Callus	PAT	*A. tumefaciens*	Particle bombardment	Herbicide resistance	[172]
*Puccinellia tenuiflora*	Cell suspension	AMT1/GFP	*A. tumefaciens*	Co-culture	Subcellular localization	[173]
	Callus	GUS, Hyg	*A. tumefaciens*	Co-culture + US + vacuum	Gene function analysis	[174]

* Gene(s): AMT1, Ammonium transporter; FLC, Flowering control gene; GFP, Green fluorescent protein; GUS, β-glucuronidase gene; Hyg, Hygromycin; NPTII, Neophosphotransferase; PAT, Phosphinothricin acetyltransferase; Ri-TDNA, Root induction TDNA.

### Somatic Hybridization

The transfer of new characters from a wild accession to cultivated crops usually starts with cross-pollination. The combination of parental genomes is also possible through somatic hybridization and enables the transfer of valuable traits through protoplast fusion and to overcome sterility or sexual incompatibility among plant species or genera. A partial transfer of organelles is also possible with the formation of cybrids. Based on the success of somatic hybridization between wheat and related intergeneric grasses, some experiments were designed to study whether salt resistance could be transferred into wheat. Xia et al. [175] published preliminary results of asymmetric fusion between *T. aestivum 5* and *L. chinensis*, a forage grass of high quality and resistant to cold, drought, salinity, and many diseases. Further analyses showed that the hybrid nature of regenerated colonies of wild *Triticum* with ultra-violet light irradiated *Leymus* protoplasts [176]. This team also regenerated fertile hybrid plants produced via somatic hybridization of protoplasts of *A. elongatum* irradiated by ultra-violet light fused with protoplasts of *T. aestivum*. Fertile intergeneric somatic hybrid plants were produced, and various asymmetric hybrid lines have been selected and propagated in successive generations. The phenotype and chromosome number of wheat could be maintained besides transfer of a few chromosomes and chromosomal fragments from the donor *A. elongatum* [177].

In another study by Wei et al. [178], protoplasts of wheat were fused with the UV-irradiated protoplasts of *A. littoralis*. The early-formed regenerated clones were identified as hybrids by chromosome, isozyme, and RAPD analysis. Their salt-tolerant ability was compared with both parents in relative growth, proline accumulation, and Na^+^/K^+^ ratio under salt stress, and was proved higher than wheat, indicating that some corresponding genes coding salt-tolerance had been transferred into the hybrids. However, only 2 from 32 clones could differentiate to weak albinos. 

## 8. Conclusions

Halophytes have been playing an increasingly important role in different areas of biotechnology, being explored as sources of food and ingredients used in cosmetics and/or health supplements, as well as in saline agriculture. This review gathered for the first-time existing information related with halophyte in vitro culture methodologies and their applications. Many reproducible protocols have been developed for micropropagation of different halophyte species, and the carried-out studies on different stages involved in micropropagation has led to considerable improvement of protocols and methods. The most common techniques comprise the micropropagation from axillary buds via node culture, micropropagation via direct or indirect shoot neoformation, caulogenesis (shoot neoformation from callus or cell suspension cultures), somatic embryogenesis, rooting, acclimatization, germplasm conservation and cryopreservation, and callogenesis and cell suspension cultures. Several explant types have been used, comprising basal shoots and nodes, cotyledons, epicotyls, inflorescence; internodal segments, leaves, roots, rhizomes, stems, shoot tips, or zygotic embryos. Moreover, due to well-controlled conditions of in vitro systems, they are being used as a tool for studying different physiological, biochemical, and molecular processes, such as functional and salt-tolerance studies, by using different methodologies such as genetic transformation (transgenesis), somatic hybridization, or androgenesis. The application of new technologies to improve halophytes will be the opportunity to improve their handling and production, aiming to obtain the desired valuable characteristics such as increased production of nutrients and metabolites, as well as resistance to salt stress.

## Figures and Tables

**Table 2:** Tissue and cell culture experiments reported in halophytes and their applications.

Family/Species	Explant Source *	Medium **	Conditions	Growth Regulators ***	Optimal Conditions	Result	Aim/Application	Reference
**Acanthaceae**								
*Acanthus ilicifolius* L.	R	MS		2,4-D, IAA, NAA, BAP, Kin	0.3 mg/L 2,4-D + 0.5 mg/L BAP	Callus	Biological activities	[136]
*Avicennia alba* Blume	C, H	AAM		2,4-D, TDZ	1 μM 2,4-D + 1 μM TDZ	Callus, Protoplasts	Salt tolerance	[137]
*Avicennia marina*	R	MS		2,4-D, IAA, NAA, BAP, Kin	0.3 mg/L 2,4-D + 0.5 mg/L BAP	Protoplasts	Salt tolerance	[138]
**Aizoaceae**								
*Mesembryanthemum crystallinum*	H	LS		Kin, 2,4-D	0.5 μM Kin + 2.3 μM 2,4-D	Cell suspension	Salt responses	[139]
*Sesuvium portulacastrum*	N	MS		BAP	20 μM BAP, then 10 μM 2,4-D + 5 μM BAP	Callus	Salt tolerance	[140]
*Trianthema triquetra* Willd.	n.s.	MS, MS/2	0–200 mM NaCl	2,4,5-T, Kin	1 mg/L 2,4,5-T + 0.1 mg/L Kin; 50 or 100 mM NaCl	Callus	Antioxidant activities	[141]
**Amaranthaceae**								
*Atriplex halimus*	C, H, IS, L, ST	MS/2, B5/2		2,4-D, Kin	S, H; B5/2 + 0.5 mg/L 2,4-D + 0.5 mg/L Kin	Callus	Micropropagation	[16]
*Salicornia europaea*	H	B5		IAA, Kin	1 mg/L IAA + 10 mg/L Kin	Callus	Salt tolerance	[128]
*Salsola baryosma* (Roem. & Schult.) Dandy (syn. *Caroxylon imbricatum* (Forssk.) Akhani & Roalson)	n.s.	MS, MS/2	0–200 mM NaCl	2,4,5-T, Kin	1 mg/L 2,4,5-T + 0.1 mg/L Kin; 0–100 mM NaCl	Callus	Antioxidant activities	[141]
*Salsola lanata*	IS, L	MS		BAP, Kin, 2iP, IBA, 2,4-D	9 µM 2,4-D	Callus	Salt tolerance	[66]
*Salsola pestifer*	IS, L	MS		BAP, Kin, 2iP, IBA, 2,4-D	8.8 µM BAP or 4.9 µM IBA	Callus	Salt tolerance	[66]
*Suaeda maritima* (L.) Dumort.	H	MS	0–400 mM NaCl	2,4-D, Kin	1 μM 2,4-D + 1 μM Kin; 0 or 200 mM NaCl	Callus	Salt tolerance	[142]
	H	B5		IAA, Kin	1 mg/L IAA + 10 mg/L Kin	Callus	Salt tolerance	[124]
*Suaeda monoica Forssk. ex J.F.Gmel.*	H	MS	0–1000 mM NaCl	2,4-D, BAP, NAA, Kin	1 mg/L 2,4-D + 0.5 mg/L BAP; 500 mM NaCl	Callus	Salt tolerance	[17]
*Suaeda nudiflora*	Ep	MS	0–1000 mM NaCl	2,4-D, BAP, NAA, Kin	0.5 mg/L 2,4-D + 0.25 Kin; 0 mM NaCl	Callus	Salt tolerance	[17]
*Suaeda salsa*	H	MS		2,4-D, BAP	0.2 mg/L 2,4-D + 0.5 mg/L BAP	Callus	Betacyanin synthesis	[143]
**Asteraceae**								
*Aster tripolium* (syn. *Tripolium pannonicum*)	C	MS		2,4-D, Zea, 2iP	0.5 mg/L 2,4-D + 0.1 mg/L Zea, transferred to 0.1 mg/L 2,4-D + 1 mg/L 2iP	Callus, cell suspension	Salt responses	[144]
*Inula crithmoides* L.	L	MS		2,4-D, IBA, NAA	1 mg/L 2,4-D	Callus	Biological activities	[145]
**Brassicaceae**								
*Cakile maritima* Scop.	IS	B5		2,4-D, Kin	9.06 μM 2,4-D + 0.46 μM Kin	Callus	Salt tolerance	[146]
		B5	0–800 mM NaCl	2,4-D, Kin	0.2 μM 2,4-D + 0.45 μM Kin	Cell suspension	Salt responses	
	n.s.	MS	50–400 mM NaCl	2,4-D	0.2 mg/L 2,4-D	Cell suspension	Salt responses	[147]
	n.s.	MS	50–400 mM NaCl	2,4-D	0.2 mg/L 2,4-D	Cell suspension	Salt responses	[148]
*Thellungiella halophila* (Bayanaul) (syn. *Eutrema halophilum* (C.A.Mey.) Al-Shehbaz & Warwick)	L	MS		2,4-D, Kin	1 mg/L 2,4-D + 0.05 mg/L Kin	Callus	Salt responses	[149]
**Clusiaceae**								
*Calophyllum inophyllum* L.	R	MS		2,4-D, IAA, NAA, BAP, Kin	0.3 mg/L 2,4-D + 0.5 mg/L BAP	Callus	Biological activities	[136]
**Euphorbiaceae**								
*Excoecaria agallocha* L.	R	MS		2,4-D, IAA, NAA, BAP, Kin	0.3 mg/L 2,4-D + 0.5 mg/L BAP	Callus	Biological activities	[136]
**Lythraceae**								
*Sonneratia alba* Sm.	Pistil	MS		2,4-D, phenylurea	0.1 µM 2,4-D + 0.1 µM Phenylurea	Callus	n.s.	[150]
		MS	0–500 mM NaCl		50 mM NaCl	Callus	Salt responses	[151]
**Malvaceae**								
*Kosteletzkya virginica* (syn. *Kosteletzkya pentacarpos*)	Callus	MS	0–255 mM NaCl		85 mM NaCl	Cell suspension	Salt tolerance	[152]
**Plumbaginaceae**								
*Armeria maritima*	C, L, R, YL	MS		2,4-D, NAA, Kin, BAP	4.5 μM 2.4-D + 0.93 μM Kin	Callus, cell suspension	Bioproduction	[153]
**Poaceae**								
*Diplachne fusca*	In	MS		2,4-D	1 mg/L 2,4-D	Callus	Salt tolerance	[45]
*Distichlis spicata*	ST	MS		2,4-D	4 mg/L 2,4-D	Callus, cell suspension	Salt tolerance	[154]
*Puccinellia tenuiflora* (Griseb.) Scribn. & Merr.	Seed	MS		2,4-D	4 mg/L 2,4-D	Callus	Salt responses	[13]
*Spartina patens*	Seedling	MS		BAP, NAA, 2,4-D, CW	0.5 mg/L 2,4-D + 0.5 mg/L BAP + 1 mg/L NAA + 5% CW	Callus	Salt tolerance	[110,155]
*Spartina pectinata* Link (syn. *Sporobolus michauxianus* (Hitchc.) P.M.Peterson & Saarela)	In	MS		2,4-D	2 mg/L 2,4-D	Cell suspension	Salt tolerance	[156]
**Rhizophoraceae**								
*Bruguiera sexangula* (Lour.) Poir.	L, seedling	MS, AAM		2,4-D, Phenylurea	AAM + 2 µM 2,4-D + 2 µM Phenylurea	Callus, cell suspension	Salt responses	[157,158]
*Ceriops decandra* (Griff.) W.Theob.	R	MS		BAP, IAA, IBA, NAA	0.5 mg/L BAP + 2.5 mg/L NAA	Callus	Bioproduction	[134]
*Rhizophora apiculata* Blume	L	MS	4–20 g/L AC	BAP, NAA	12 g/L AC; 0.3 mg/L BAP + 1 mg/L NAA	Callus	n.s.	[159]
**Salvadoraceae**								
*Salvadora persica*	N	MS		2,4,5-T, BAP	0.5 mg/L 2,4,5-T + 0.5 mg/L BAP	Callus	Salt responses	[160]
**Zygophyllaceae**								
*Nitraria tangutorum* Bobr.	C	MS		NAA, BAP	0.3 mg/L BAP + 1 mg/L NAA	Callus	Salt responses	[161]
*Tetraena simplex* (L.) Beier & Thulin	n.s.	MS, MS/2	0–200 mM NaCl	2,4,5-T, Kin	0.5 mg/L 2,4,5-T + 0.1 mg/L Kin; 50 and 100 mM NaCl	Callus	Antioxidant activities	[141]

n.s.: non specified; * Explant sources: BN—basal node; BS—basal shoot; C—cotyledon; Ep—epicotyl; H—hypocotyl; In—inflorescence; IS—internodal segment; L—leaf; N—node; R—root; Rh—rhizome; S—stem; ST—shoot tip; ZE—zygotic embryo. ** Basal medium—B5—Gamborg medium [119]; H&A—Hoagland and Arnon medium [120]; LS—Linsmaier and Skoog medium [162]; MS—Murashige and Skoog medium [121]; WPM—Woody plant medium [122]. *** Growth regulators: 2iP—2-Isopentenyl adenine; 2,4-D—2,4-Dichlorophenoxyacetic acid; 2,4,5-T—2,4,5-Trichlorophenoxyacetic acid; AdS—Adenine sulfate; BAP—6-Benzylaminopurine; CNH—Casein hydrolysate (casaminoacids); CPA—4-Chlorophenoxyacetic acid; CW—Coconut water; GA3—Gibberellic acid; Gln—Glutamine; Glu—Glutamic acid; IAA—Indole-3-acetic acid; IBA—Indole-3-butyric acid; IPA—Indole-3-propionic acid; Kin—Kinetin; NAA—1-Naphtaleneacetic acid; PGRs—Plant growth regulators; TDZ—Thidiazuron (1-phenyl-3-(1,2,3-thiadiazol-5-yl) urea; Zea—Zeatin.

## Data Availability

Data is contained within the article.

## List of Abbreviations

2,4-D	2,4-Dichlorophenoxyacetic acid
2,4,5-T	2,4,5-Trichlorophenoxyacetic acid
BAP	6-Benzylaminopurine
Bl	Brassinolides
CMCs	Cambial meristematic cells
CNH	Casein hydrolysate (casaminoacids)
CW	Coconut water
Gln	Glutamine
Glu	Glutamic acid
H&A	Hoagland and Arnon
IAA	Indole-3-acetic acid
IBA	Indole-3-butyric acid
IPA	Indole-3-propionic acid
Kin	Kinetin
MeJ	Methyl jasmonate
LS	Linsmaier and Skoog
MS	Murashige and Skoog
mT	meta-Topoline
n.s.	Non-specified
NAA	1-Naphthaleneacetic acid
PAL	Phenylalanine ammonium lyase
PGRs	Plant growth regulators
SE	Somatic embryogenesis
SMs	Secondary metabolites
SOS	Salt Overly Sensitive
TDZ	Thidiazuron (1-phenyl-3-(1,2,3-thiadiazol-5-yl) urea)
WPM	Woody plant medium
ZEA	Zeatin
